# Scale-Free Functional Connectivity of the Brain Is Maintained in Anesthetized Healthy Participants but Not in Patients with Unresponsive Wakefulness Syndrome

**DOI:** 10.1371/journal.pone.0092182

**Published:** 2014-03-19

**Authors:** Xiaolin Liu, B. Douglas Ward, Jeffrey R. Binder, Shi-Jiang Li, Anthony G. Hudetz

**Affiliations:** 1 Department of Biophysics, Medical College of Wisconsin, Milwaukee, Wisconsin, United States of America; 2 Department of Neurology, Medical College of Wisconsin, Milwaukee, Wisconsin, United States of America; 3 Department of Anesthesiology, Medical College of Wisconsin, Milwaukee, Wisconsin, United States of America; Hangzhou Normal University, China

## Abstract

Loss of consciousness in anesthetized healthy participants and in patients with unresponsive wakefulness syndrome (UWS) is associated with substantial alterations of functional connectivity across large-scale brain networks. Yet, a prominent distinction between the two cases is that after anesthesia, brain connectivity and consciousness are spontaneously restored, whereas in patients with UWS this restoration fails to occur, but why? A possible explanation is that the self-organizing capability of the brain is compromised in patients with UWS but not in healthy participants undergoing anesthesia. According to the theory of self-organized criticality, many natural complex systems, including the brain, evolve spontaneously to a critical state wherein system behaviors display spatial and/or temporal scale-invariant characteristics. Here we tested the hypothesis that the scale-free property of brain network organization is in fact fundamentally different between anesthetized healthy participants and UWS patients. We introduced a novel, computationally efficient approach to determine anatomical-functional parcellation of the whole-brain network at increasingly finer spatial scales. We found that in healthy participants, scale-free distributions of node size and node degree were present across wakefulness, propofol sedation, and recovery, despite significant propofol-induced functional connectivity changes. In patients with UWS, the scale-free distribution of node degree was absent, reflecting a fundamental difference between the two groups in adaptive reconfiguration of functional interaction between network components. The maintenance of scale-invariance across propofol sedation in healthy participants suggests the presence of persistent, on-going self-organizing processes to a critical state – a capacity that is compromised in patients with UWS.

## Introduction

An essential feature of the healthy brain is that anesthetic-induced loss of consciousness in general anesthesia is spontaneously and completely reversible after the withdrawal of the anesthetic. Spontaneous restoration of consciousness is, however, absent in patients with UWS (also known as persistent vegetative state). During the past two decades, numerous neuroimaging studies have been conducted to determine neural correlates of unconsciousness in general anesthesia, coma, vegetative state, sleep, and seizure, particularly focusing on the integrity and reversibility of large-scale functional networks of the brain [1], [2], [3], [4], [5], [6]. Studies conducted in unconscious conditions of different etiology generally showed common changes in regional cerebral metabolic rate and in functional connectivity measured by the temporal correlation of neurophysiological events between spatially distinct brain regions [7], [8], [9]. Given the similarity of functional alterations in various unconscious conditions, a critical question is whether there is a further aspect of network organization that allows the healthy brain to recover consciousness, for example, after general anesthesia, but whose absence impedes the recovery in severe neuropathological conditions, such as UWS.

One possible explanation is that for the healthy brain, the spontaneous restoration of consciousness after anesthesia is a consequence of the self-organizing ability of neural networks, which allows organization towards wakeful baseline consciousness to occur without any predefined plans or external manipulation of system parameters. Conversely, it is possible that this self-organizing capability is compromised or absent in patients with UWS. The purpose of this study was to examine differences in network characteristics related to the self-organizing ability of the brain in anesthetized healthy participants and in patients with UWS.

A theoretical framework for explaining the emergence of complexity and ubiquitous scaling laws in nature proposes that complex systems often maintain a state of self-organized criticality (SOC) [10], [11], i.e., that they self-organize to operate near a critical point of phase transitions. The theory was initially illustrated by a sand-pile model. When a sand pile is flat, dropping sand to the sand pile only cause local arrangement to occur, with no self-organization and interesting dynamics. In contrast, when a sand pile is too steep, it will collapse by itself until the average slope reaches a critical value where the system is minimally stable with respect to small perturbations. A sand pile thus always tends to evolve to and maintain a critical slope or a critical state. Dropping sand to a sand pile in a critical state will cause reorganization of sand to occur across all scales (or without a characteristic scale, i.e., scale invariance), which will ultimately evolve to a new critical slope after equilibrium is reached. The central message of the theory of SOC suggests that many types of natural complex systems may approach a critical state through self-organization, which naturally giving rise to power-law correlation functions for noise and other observable physical quantities. By this framework, the concepts of criticality, self-organization, and power-law (scale-free or fractal) scaling characteristics of a complex system are linked with each other [12]. Many real-world systems including earthquakes, forest fires, mountain avalanches, and heartbeat rhythms are now recognized to exhibit critical dynamics. Power-law or scale-free scaling has been considered an empirical signature of complex, non-equilibrium systems in a self-organized critical state [11].

In neuroscience, it has been suggested living systems or the working brain self-organize to operate near a critical point [13], [14], [15]. A complex systems operating in a critical state is optimal in performing various functions including system responsiveness, adaptability, information transfer and storage, etc. [16]. Although direct evidence of criticality in the brain is sparse, and the set of general characteristics to guarantee that a system is in SOC remains to be defined [12], [13], the presence of scale-free characteristics in space or time has been found across all levels of nervous systems [13], [14], [17], [18]. Neuronal avalanches recorded by multielectrode array follow a scale-free distribution and suggest the presence of critical dynamics in the brain [15], [16], [19], [20], [21], [22], although the issue is still under debate [23]. Phase synchronization and liability of global synchronization of the resting-state whole-brain functional network in healthy volunteers measured by functional magnetic resonance imaging (fMRI) and magnetoencephalography (MEG) demonstrate power-law scaling consistent with predictions by computational models, such as the Ising model and Kuramoto model [21]. In addition, large-scale brain network architecture identified by MEG [21], [24], electroencephalography (EEG) [25], and fMRI [26], [27], [28] exhibit scale-free properties in conscious healthy human subjects.

Given the connection between the ability of a complex system to self-organize to a critical state and the manifestation of scale-free properties, and the proposition that consciousness emerges from brain function as a network phenomenon [8], [9], here we test two hypotheses: (1) scale-free brain network organization is present in healthy awake humans and is preserved during anesthesia-induced unconsciousness; (2) scale-free brain network organization is in some way disrupted in patients with UWS. These hypotheses were formulated based on the presumed relationship between the self-organizing capability of the brain and spontaneous recovery of consciousness as well as the known facts that healthy subjects are able to regain consciousness after anesthesia but patients with UWS fail to do so. To date, few studies have investigated potential alterations in scale-free properties of brain network organization during loss of consciousness in healthy or pathological brain conditions. Using multichannel EEG data, Lee et al. found that global scale-free organization of the brain was maintained across different states of consciousness modulated by the anesthetic propofol [29]. Achard et al. investigated using resting-state fMRI the functional connectivity characteristics of brain network organization in 17 comatose patients who were scanned a few days after major acute brain injury [30]. They reported that global topological properties were similar in comatous patients compared to healthy controls, but major network hubs were significantly reorganized in the patients. In addition, the node-degree (number of links per network node, usually above a threshold) distribution exhibited an exponentially truncated power law in both patients and healthy controls. However, the population was relatively mixed, as five patients were diagnosed with UWS six months later, while another three patients recovered and nine patients died. Thus, it is not clear from the pooled results and delayed diagnosis if in patients with UWS, scale-free node-degree distribution was present or not.

Detection of scale-free properties in brain network organization seems to be affected by the way network nodes are defined. At voxel resolution, a handful of fMRI studies revealed scale-free organization as indicated by power-law-distributed node degree in healthy awake humans [27], [28], [31]. Other fMRI studies that used anatomical parcellation of brain regions (e.g., 90 nodes) found instead an exponentially truncated power-law node-degree distribution [26], [32]. It is possible that coarse-grained anatomical parcellation may ignore distinct functional nodes contained within each of the individual anatomical structures. In contrast, the voxel-based approach may result in too many redundant network nodes that have a nearly identical hemodynamic response profile on fMRI.

In this study, we introduce a new combined anatomical-functional parcellation algorithm that identifies distinct functional nodes within each of the individual anatomical structures defined by a standard template in the Talairach space [33]. Specifically, we define network nodes as clusters of voxels that share a similar blood-oxygen-level-dependent (BOLD) time series profile in one run but are spatially confined by the boundaries of an individual anatomical structure. This clustering process was first performed separately with each of the 116 standard anatomical structures [33] by taking account of all voxel time series included in an anatomical structure. The final number of voxel clusters (network nodes) formed within an individual anatomical structure was then determined by applying a global threshold to the hierarchical linkage-distance tree generated with each anatomical structure. By varying the global threshold, the whole-brain network can be defined flexibly at any desired spatial scale, or equivalently, at any total number of network nodes. As we will show, this balanced anatomical-functional parcellation approach with flexibility in controlling the spatial scale of a defined network is capable of revealing scale-free network organizations as the number of network nodes are defined at sufficiently fine scales. Such a capacity plays an essential role in differentiating brain network organizations of anesthetized healthy participants from those of patients with UWS.

## Materials and Methods

### Data acquisition

The fMRI data analyzed by this study were published in our two previous investigations of healthy human participants undergoing propofol sedation [6], [34] and patients with UWS [5]. Healthy participants in the fMRI propofol sedation study provided written informed consent to participate. Experimental protocols were approved by the Institutional Review Board of the Medical College of Wisconsin (Milwaukee, WI). Informed written consent was obtained from the families of patients with UWS for the resting-state fMRI scans. Experimental protocols were approved by the Ethics Committee of Capital Medical University (Beijing, China). The patients were diagnosed with UWS after repeated clinical tests using the standard Glasgow Coma Scale (GCS) and the Chinese Vegetative State Scale (CVSS). Clinical profiles of the UWS patients reported in this study are summarized in Table 1 and also in the previous publication [5].

**Table 1:** Clinical profiles of the five patients with UWS reported in this study.

Patient	Diagnosis	Age	G	Time (d)	GCS		CVSS
1	hydrocephalus, L frontal Contusion, SAH (subarachnoid hemorrhage)	32	M	42		9	8
2	Bi frontal and temporal contusion	24	M	63		8	6
3	R temporal and frontal contusion, thalamus hemorrhage	45	M	46		10	9
4	Bi frontal and R occipital contusion	48	M	59		10	9
5	L temporal and parietal hemorrhage	43	F	66		8	7

(L  =  left, R  =  right, Bi  =  bilateral).

In the propofol study, blood oxygen level-dependent (BOLD) signals at 1.5 Tesla (repetition time, 2s; in-plane resolution, 3.75×3.75 mm; thickness, 6 mm) were collected from eight healthy volunteers (four men and four women; aged 24 to 42; body mass index <25) who performed a verbal memory task in the scanner during each of the three 6-minute-long fMRI runs in wakeful baseline, propofol-induced deep sedation, and recovery. There were about 15 minutes of separation between the runs for experimental preparation. During deep sedation, the target plasma concentration of propofol was set as 2 μg/ml; at this anesthetic depth, the participants no longer responded to verbal commands. The resting-state BOLD signal acquisition in five patients with UWS was performed at 3 Tesla [5] (duration of 6 minutes; repetition time, 2s; in-plane resolution 3.75×3.75 mm; slice thickness, 5 mm).

### Data preprocessing

Imaging data analysis was conducted using Analysis of Functional NeuroImages (AFNI, http://afni.nimh.nih.gov/afni) and Matlab (The MathWorks, Natick, MA) software. For both sets of data, high-resolution anatomical images obtained during the scan session were transformed into standard Talairach space, followed by co-registration of the functional data to the Talairach space with resampling to 2-mm cubic voxels (*adwarp* in AFNI). Subsequent data preprocessing included despiking, detrending (*3dDetrend* in AFNI, using the Legendre polynomials with an order of 3) and motion correction (*3dvolreg* in AFNI, producing three translational and three rotational parameters for each image). The first four points of the voxel time series of each run were discarded to reduce the initial transient effects. Physiological noise contributions from the white matter and the cerebrospinal fluid were estimated using the average BOLD signal time series of these structures, which were manually identified in each individual's anatomical images. The voxelwise BOLD time series from each scanning run were then analyzed with a general linear regression model (*3dDeconvolve* in AFNI) using the eight regressors representing noise artifacts from the six motion parameters, white matter, and cerebrospinal fluid. The residual signals of the regression analysis were considered representative of the denoised voxelwise BOLD time series.

### Network partitioning and evaluation of scale-free statistics

**Figure 1:**
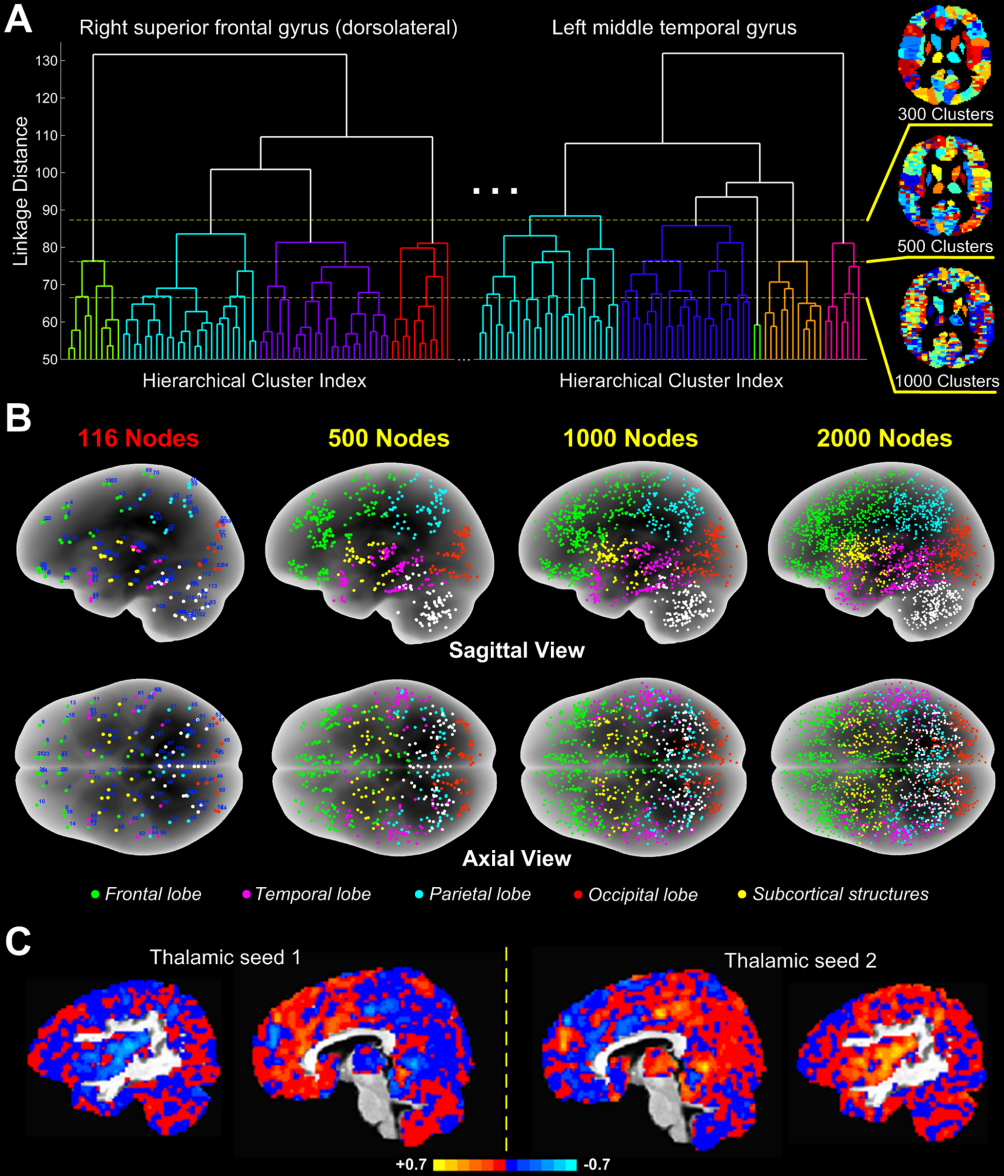
A combined anatomical-functional parcellation algorithm that defines nodes of the whole-brain network at an arbitrary scale. **(A)** The algorithm involves performing a hierarchical clustering analysis with all voxels included in each of the 116 individual anatomical structures, followed by applying a global threshold to the generated linkage-distance tree with each single anatomical structure to separate voxel clusters functionally distinct from each other. The panel A shows the thresholds at three different values that divide the whole brain into 300, 500, and 1000 clusters (nodes). The color-coded cluster distributions were shown at an intermediate slice of the axial plane. **(B)** The centers of mass (nodes) of the 116 anatomical structures (from the standard template) and of voxel clusters obtained using procedures described in A at three different spatial scales are marked by distinct colors according to their lobe or subcortical location. The number and distribution of network nodes are approximately equal in the left and right hemispheres across the spatial scales. **(C)** Full correlation maps computed using two identified thalamic voxel clusters as the seeds for connectivity analysis showed largely opposite patterns of correlation magnitude in the overall distribution pattern and in local details. One lateral and one medial slices along the sagittal plane were shown for the two maps (*left* and *right* panels).

The constituent nodes of the whole-brain network were defined as clusters of voxels sharing a similar hemodynamic profile in one run and spatially confined within the boundaries of a single anatomical structure. The choice to use anatomically constrained functional parcellation was motivated by the hierarchical modular organization of human brain networks [35], [36] and the fact that the neuroanatomical substrate provides the structural basis for the distribution, stability, and diversity of the basic functional units of brain networks. [37], [38]. Our anatomical-functional parcellation scheme consisted of the following steps (Figure 1A). First, 116 anatomical regions in Talairach space were delineated in each subject's anatomical images according to a standard reference template [33] (tt_n27_ez_ml.tlrc, provided with AFNI software package). Second, hierarchical clustering was conducted separately each time with the preprocessed BOLD time series of all voxels included in each of the 116 anatomical structures. The clustering was based on computing a dendrogram using the inner squared distances (the minimum variance) among the normalized voxel time series, resulting in a hierarchical linkage distance tree for each anatomical structure. Third, we applied a global threshold of linkage distance to all obtained 116 dendrograms to determine the number of clusters formed within each of the anatomical structures, and therefore the total number of network nodes. The magnitude of global threshold is solely determined by the spatial scale, at which a desired number of clusters (nodes) are obtained to cover the entire brain. In this study, the number of network nodes ranges from 116 (the original anatomical partitions) up to 4000 with step increases across different spatial scales. To better describe the changes in the results, the step increase of the number of network nodes is finer at coarse spatial scales than at relatively fine scales. For each fMRI run, the mean voxel time series of each identified voxel cluster was considered representative of the node time series used in node degree (connectivity) analysis.

After defining network nodes at a given spatial scale, the cross-correlation between each pair of the nodes was computed using the node time series, resulting in a symmetric cross-correlation matrix with a dimension equivalent to the total number of network nodes. Histograms were then computed to evaluate two essential properties of the brain network organization: (1) the distribution of the size of network nodes measured by the number of voxels contained in each node and (2) the distribution of the number of node connections (node degree) after thresholding the cross-correlation matrix. The threshold of the cross-correlation matrix was set at 0.2 for the BOLD signals acquired at 1.5 Tesla with healthy participants and at 0.4 for the BOLD signals acquired at 3.0 Tesla with UWS patients. The shapes of histograms were then evaluated according to their fit to a power-law distribution. The goodness-of-fit was evaluated by conducting a linear regression with logarithm-transformed histogram data at a representative spatial scale (2000 network nodes in this report). To assess whether that the power-law distribution provides the best fit of the data, we compared the fit of the power-law model with those of exponential and logarithm models in terms of mean squared error (MSE). These two additional models were chosen because they are the most common models that could potentially provide a good fit to data of similar shape to a power-law distribution. The comparisons were conducted by paired two-sample *t*-tests with results considered significant at *p* <0.05.

## Results

Network nodes within each hemisphere exhibited similar distributions with respect to their total number (e.g., at the scale of 2000 network nodes: Left, 1007±46; Right, 993±46; *P* = 0.67 for a significant difference) and average size (Left, 71.5±3.4; Right, 72.4±3.2; *P* = 0.72) in healthy participants during wakefulness (Figure 1B). Moreover, the spatial locations of network nodes generally showed a trend of interhemispheric symmetry across examined spatial scales. These characteristics were also replicated in the states of deep sedation and recovery.

To verify whether the identified voxel clusters (nodes) truly capture functionally distinct units within each of the individual anatomical structures, we performed a connectivity analysis using eight voxel clusters identified from the whole thalamus as seed regions. The unthresholded full correlation maps derived from two seed regions were shown at a same sagittal slice (Figure 1C, *left* and *right* panels). Compared with each other, the correlation maps revealed largely opposite patterns of correlation magnitude in the overall distribution pattern and in local details, indicating that the combined anatomical-functional parcellation algorithm is capable of differentiating hemodynamically distinct voxel clusters within an anatomical structure.

### Node-size distribution in wakefulness, deep sedation, and recovery

**Figure 2:**
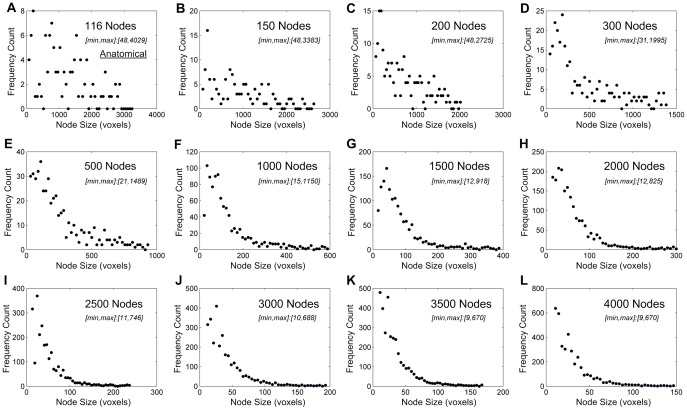
Node-size distribution across different spatial scales in one healthy participant at the wakeful baseline. (**A**) Node-size distribution of the original 116 anatomical nodes. (**B-L**) As network nodes were defined at finer spatial scales, a power-law node-size distribution became increasingly evident. The minimum and maximum node sizes were shown in the subplots.

**Figure 3:**
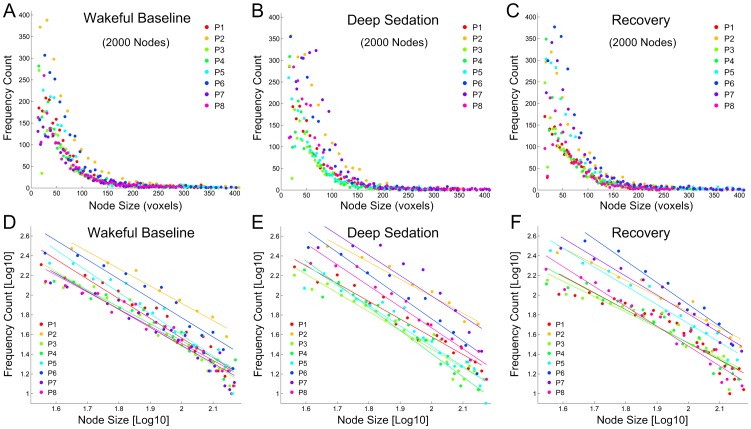
Node-size distributions at the spatial scale of 2000 nodes in eight healthy participants in the states of wakeful baseline, deep sedation, and recovery. **(A-C)** Node-size distributions in the three states of consciousness. **(D-F)** Linear fittings of logarithm-transformed frequency count and node size. Power-law fitting is significantly better or demonstrates a stronger trend than exponential and logarithmic fittings in minimizing MSE, providing a better fit of the data.

In all three conditions, a power-law distribution of node size became evident as the number of network nodes was increased. As an example from one of the participants, a power-law node-size distribution at wakeful baseline was first recognizable at 300 nodes and became increasingly evident at higher numbers of node partitions (Figure 2). Similar effects were present in deep sedation and recovery (Figure S1 and Figure S2). Across all subjects, power-law distributions of node size and degree were clearly present at and above 2000 nodes. We therefore chose the 2000-node parcellation as a representative spatial scale for the demonstration of group results. Node-size distributions at this spatial scale are summarized for the eight participants across the three states of consciousness in Figure 3A-C, with linear regression of logarithm-transformed data (within the range of node sizes from about 60 to 280 voxels) in Figure 3D-F. The r-squared values for power-law fitting were 0.95±0.02, 0.94±0.03 and 0.93±0.03 for the states of wakefulness, deep sedation, and recovery, indicating a good fit of the data. Compared with exponential and logarithmic models, power-law fitting was significantly better or demonstrated a stronger trend in minimizing MSE, suggesting a better fit overall (Table 2).

**Table 2:** MSE for three models of node-size distribution in the three states of consciousness.

	Power-law fit	Exponential fit	Logarithmic fit	P-value (P<E)	P-value (P<L)
Wakefulness	55±37	96±78	158±127	0.04	0.01
Sedation	74±54	85±113	138±124	0.37	0.06
Recovery	119±86	135±213	250±337	0.39	0.1

(P: Power law fit; E: Exponential fit; L: Logarithmic fit).

### Node-degree distribution in wakefulness, deep sedation, and recovery

**Figure 4:**
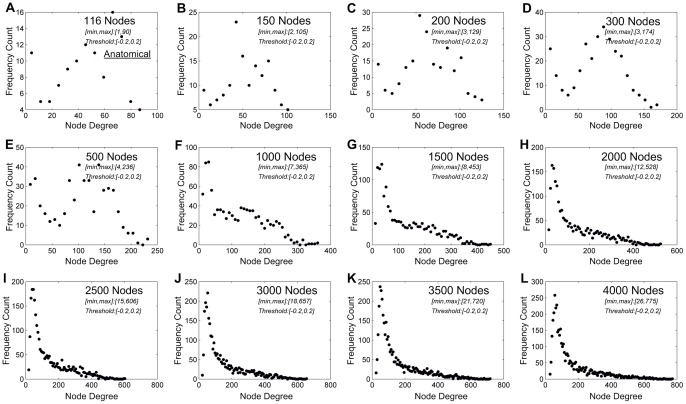
Node-degree distribution in one healthy participant (as shown in Figure 2) at the wakeful baseline. (**A**) Node-degree distribution of the original 116 anatomical nodes. (**B-L**) As network nodes were defined at finer spatial scales, a power-law node-degree distribution became increasingly evident. The minimum and maximum node degrees after thresholding were shown in the subplots.

**Figure 5:**
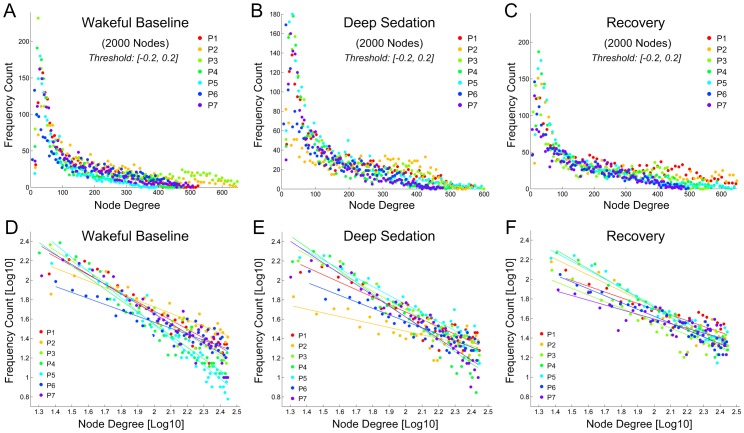
Node-degree distributions at the spatial scale of 2000 nodes in seven healthy participants in the states of wakeful baseline, deep sedation, and recovery. **(A-C)** Node-degree distributions in the three states of consciousness. **(D-F)** Linear fittings of logarithm-transformed frequency count and node degree. Power-law fitting is significantly better than exponential and logarithmic fittings in minimizing MSE, providing a better fit of the data.

As with node-size distribution, a power-law distribution of node degree became evident as the number of network nodes was increased. As demonstrated in data from the same participant as shown in Figure 2, the node-degree distribution followed a power-law distribution at 2000 nodes or higher (Figure 4). In seven of eight participants, node-degree distribution followed a power-law at or above 2000 nodes. One participant's data showed an abnormal pattern of connectivity in deep sedation possibly due to noise of unknown origin during scanning and were excluded from this analysis. The same pattern of power-law node-degree distribution was observed during deep sedation (Figure S3) and recovery (Figure S4). The node-degree distributions at the spatial scale of 2000 nodes are summarized for the seven participants across the three states of consciousness in Figure 5A-C, with linear regression of logarithm-transformed data (within the range of node degree from about 20 to 280 connections) in Figure 5D-F. The r-squared values for power-law fitting were 0.9±0.05, 0.85±0.1 and 0.9±0.07 for the states of wakefulness, deep sedation, and recovery, indicating a good fit of the data. Compared with exponential and logarithmic fittings, power-law fitting is significantly better in minimizing MSE in all three states, suggesting a better fit of the data (Table 3).

**Table 3:** MSE for three models of node-degree distribution in the three states of consciousness.

	Power-law fit	Exponential fit	Logarithmic fit	P-value (P<E)	P-value (P<L)
Wakefulness	128±112	455±284	270±171	0.004	0.01
Sedation	76±58	210±125	127±73	0.000	0.02
Recovery	82±52	285±157	155±85	0.004	0.009

(P: Power law fit; E: Exponential fit; L: Logarithmic fit)

### Node-size and node-degree distributions in UWS patients

**Figure 6:**
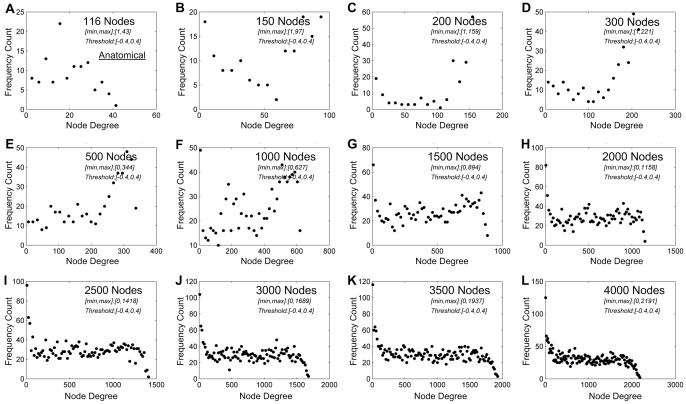
Node-degree distribution in one example of the UWS patients. (**A**) Node-degree distribution of the original 116 anatomical partitions. (**B-L**) Regardless of spatial scale at which a whole-brain network was defined, the UWS patient showed no power-law-shaped node-degree distribution as compared to that observed in healthy individuals.

**Figure 7:**
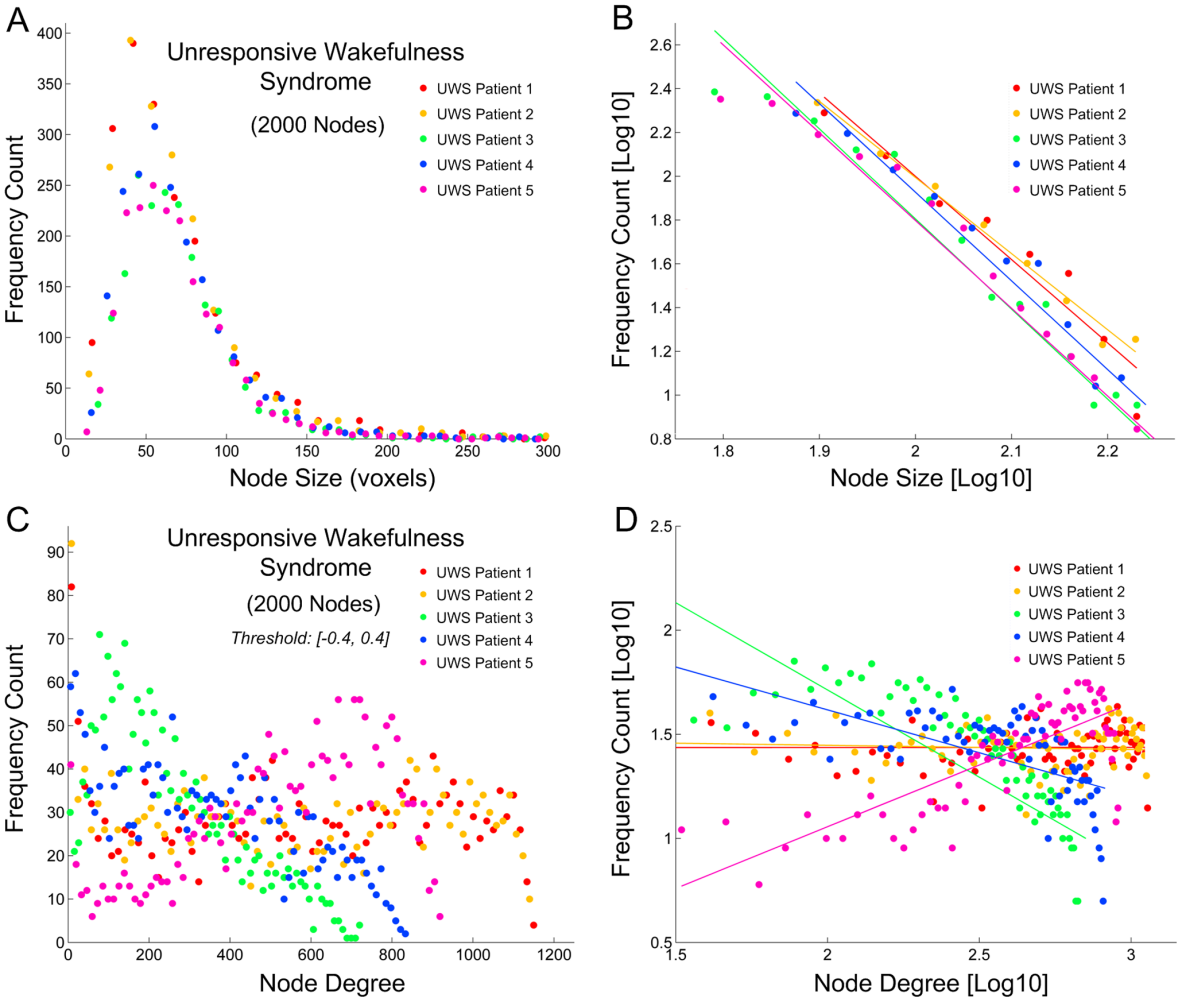
Node-size and node-degree distributions in five UWS patients. (**A-B**) Power-law-shaped node-size distributions were maintained in (A), with linear fittings of logarithm-transformed frequency count and node size in (B). (**C-D**) In contrast to that in healthy individuals (Figure 5), node-degree distributions in UWS patients demonstrated no evidence of a power-law relation (C), with linear fittings of logarithm-transformed frequency count and node degree showing inconsistent profiles (D).

Node-size distribution in patients with UWS showed a consistent a power-law-shaped distribution at approximately the spatial scale of 2000 nodes (Figure S5). However, unlike the healthy sedated individuals, patients with UWS failed to show a power-law-shaped node-degree distribution even at fine-scale node parcellations, regardless of threshold. One example from the UWS patients demonstrated a nearly flat node-degree distribution across all fine spatial scales (Figure 6). The node size and degree distributions at the spatial scale of 2000 nodes are summarized for the five UWS patients in Figure 7. It is obvious from the figure that the node-degree distribution varied significantly across the patients, but none of them adheres in general to the profile of a power-law distribution.

## Discussion

Understanding the neurobiological mechanisms that govern the state of consciousness as modulated in physiological, pharmacological or pathological conditions is arguably one of the most fundamental challenges in neuroscience. The power of the brain to self-organize may be particularly relevant to the subject of consciousness [39], specifically with respect to the question of why consciousness spontaneously recovers in the healthy brain after general anesthesia or deep sleep, but does not in brains under severe neuropathological conditions. The current study tested the hypothesis that the scale-free property of network organization – an empirical signature of self-organization associated complex systems in a critical state – is fundamentally different between the brains of anesthetized healthy participants and patients with UWS. As we showed in this study, the sharp contrast in scale-free characteristics of node-degree distribution between the two groups suggests that unlike the brains of anesthetized healthy humans, the brains of patients with UWS may in fact have compromised ability to self-organize and thus fail to regain consciousness.

Loss of consciousness is accompanied by significantly altered metabolic activity and functional connectivity in widespread cortical and subcortical networks in anesthetized healthy humans and patients with UWS. In both cases, prominent reductions in regional cerebral metabolism and blood flow were identified in significantly overlapping brain areas, including the prefrontal, premotor and parietotemporal association areas, the posterior cingulate and retrosplenial cortices and the precuneus [7], [9], [40], [41], [42]. In addition, the unconscious conditions are usually associated with a disruption of functional connectivity in large-scale brain networks, specifically those of the frontoparietal association cortices and the thalamocortical networks [1], [5], [6], [9], [43], [44]. Moreover, in both conditions, stimulus-dependent brain activation was found preserved in primary sensory cortices but not in higher multimodal association areas, given the overt suppression of cognitive perception [3], [34], [45], [46], [47]. These studies together suggest that changes in the area and magnitude of brain metabolic activity and functional connectivity alone are insufficient in explaining why consciousness is recoverable to anesthetized healthy humans but not to patients with UWS. Instead, these findings imply that there may be further unidentified mechanisms of brain network organization that underlie the difference between the two groups in regaining consciousness from an unconscious state.

Experimental and theoretical advances that characterize emergent properties of complex systems provide useful clues for identifying such mechanisms. According to the theory of self-organized criticality [10], complex systems evolve autonomously to a critical state characterized by power-law or scale-free characteristics, even given exogenous perturbations. There is now increasing evidence that such processes may be observed in the human brain, which is a typical example of a complex network [13], [14], [15], [16], [17], [18]. Understanding the pervasiveness and implication of scaling laws in cognitive science is still at its early stage [12], [14]. Arguments have been made that existence of scaling laws may be uninformative because of the existence of other non-critical, artificial solutions that could produce scaling laws. For example, it is easy to generate apparent power-law-looking distributions as the sum of Gaussian processes acting over different time/space scales. One specific such example is represented by the Perlin noise [48], which, however, has been widely used to simulate realistic-looking visual effects like clouds, textures, and terrains that are formed as a result of self-organization in natural complex systems. With regard to the debate, Kello et al. argued that the extent and magnitude of identified scaling laws in biology is beyond being mere coincidence, suggesting that “scaling laws describe a fundamental order in living and complex systems” [14]. Although our study was not designed and intended to resolve the debate over the presence of critical dynamics in the brain [23], experimental and theoretical advances in the fields of nonlinear dynamics, complex systems, and modern network theory do provide useful intellectual frameworks for understanding the emergent properties of human consciousness in various conditions. For example, it has been proposed that at a critical point of anesthetic-induced transition to unconsciousness, the abrupt change in brain state can be characterized by a first-order phase transition model in the cortex, similar to thermodynamic phase changes in classical physics [49], [50]. From our perspective, it is not that the presence of criticality would necessitate the presence of conscious awareness, since criticality is most likely also present during unconsciousness in the healthy brain during deep dreamless sleep or anesthesia, but that the self-organizing capability of a complex system operating in the critical regime would endow the brain with the potential to maintain or regain consciousness as it does, for example, during general anesthesia, after the withdrawal of the anesthetic, or during natural sleep under the stimulation of wake-promoting neural circuitry. Based on findings of our own and of others [13], [15], we surmise that for the healthy brain, criticality persists in both conscious and unconscious conditions, permitting transitions of the state of consciousness as produced by anesthetic administration or endogenous sleep modulation, which in turn manifest power laws. Such presence of criticality and self-organization, at least with respect to conscious functioning, is, however, compromised in patients with UWS. From this perspective, the SOC theory provides a plausible explanation for the findings of our study and therefore offers a useful conceptual framework for an understanding of emergent behaviors of human consciousness.

A novel aspect of our study is the introduction of a combined anatomical-functional parcellation algorithm that is able to define the whole-brain network across arbitrary spatial scales, representing an extension to the existing methods [51]. Macroscopic anatomical boundaries have a general, though imperfect, relation to functional boundaries. The proposition of nested network organization in the central nervous system suggests that the basic functional units of the brain first emerge individually within each of the individual anatomical structures [52], [53], [54]. Thus, by defining a network node as a cluster of voxels sharing a similar BOLD time series profile and constrained by the boundaries of a single anatomical structure, our approach takes into consideration both the anatomical and functional significances of identified network nodes, with increased node specificity (as compared with coarse-grained anatomical parcellation) and reduced node redundancy (as compared with voxel-based approach). In fact, our results show that in healthy participants, the scale-free node size and degree distributions only become evident with an increase of number of network nodes, that is, at increasingly finer spatial scales, the constitution of the most basic functional units of the brain and their interactions are getting more and more reflected by the constructed network, as suggested by Figure 1C. One could argue that if the criticality hypothesis holds true to the functioning of the brain, then the associated scale-free network characteristics should only be revealed when the structure and functional dynamics of network components can be expressed at a sufficient level of temporal and spatial resolutions. Moreover, of a noteworthy result in this study, power-law node-degree distribution, which reflects the organization of functional interactions between network nodes, appears to be more susceptible to brain injuries in the UWS patients than that of node size. We consider that to some extent the phenomenon is analogous to disruption of air traffic across the country by a large-scale severe weather condition. In such a case, the original power-law-distributed airport size would still be maintained, but the power-law-distributed airport traffic volume would be disrupted.

The preservation of scale-free distributions of node size and node degree during sedation in healthy participants is consistent with previous findings obtained with EEG [29]. Despite significant propofol-induced alterations of functional connectivity in task-related [34] and thalamocortical [6] networks, scale-free network organization was persistently maintained in propofol sedation and in subsequent recovery of consciousness with healthy participants, consistent with the prediction by the theory of self-organized criticality [10]. The findings are also compatible with the preserved fractal small-world organization obtained from MEG measurements in healthy humans in both resting and task conditions [24]. Fractal network structure naturally gives rise to scale-free characteristics [12], [13]. In both healthy humans [55], [56] and animals [57], significant spatiotemporal reconfiguration in large-scale brain functional networks during anesthetized states have been observed; nevertheless, major topological features such as small-worldness are maintained in the anesthetized brain. Also, in chronic neuronal degenerative pathologies such as the Alzheimer's disease [58], [59], the small-world characteristic of the brain remains present in spite of detectable changes in network organization. Compared with patients with chronic neuronal degenerative disease, patients with UWS are characterized by an overwhelming loss of brain functioning and consciousness. A goal of future studies may be to determine whether the loss of power-law node-degree distribution is uniquely associated with severe neuropathological conditions such as UWS. Nevertheless, with respect to the node-degree distribution in patients with UWS, our results are at variance with a recent neuroimaging study [30]. Achard et al. reported that node-degree distribution conformed to an exponentially truncated power law in both comatose patients and healthy controls, whereas our UWS patients failed to display a power-law node-degree distribution. The difference may be attributed to a few factors. First, Achard et al. scanned 17 comatose patients a few days after major acute brain injuries. Of the reported patients, five were diagnosed with UWS six month later (three other patients recovered and nine died). Because of the mixed patient population, one cannot determine whether the node-degree distribution was scale free in patients with UWS. It is likely that in coma patients, brain organization is different at the early stage of brain injury from that several months later when formal diagnosis was performed. Second, the network construction by the authors was based on 417 homogeneous anatomical partitions. In contrast, we identified functionally distinct network nodes within individual anatomical structures at a parcellation into 2000 nodes. Finally, the authors performed connectivity analysis only within a very limited frequency interval of fMRI signal (0.02–0.04 Hz) using wavelet analysis. In contrast, we did not apply a band-pass filter in order to preserve the full spectrum of the fMRI signal. Recent studies suggest that there may be important contributions from the BOLD signal at frequencies higher than the usual 0.1 Hz cutoff to the resting-state connectivity [60], [61].

Cautions in interpreting the results are in order. First, the notion and function of self-organization are only loosely defined in the literature. In this paper, we focus on self-organization in the context of the brain's ability to spontaneously recover consciousness. It should be noted that the presence of sleep-wake cycles in patients with UWS suggests a certain degree of self-organization, including the maintenance of autonomic functions by key subcortical structures, especially the brainstem [62]. Thus, there may be multiple, parallel self-organizing processes simultaneously ongoing in the brain that serve different functional goals at any one time or in a particular state. The necessity or sufficiency of criticality for consciousness in general is difficult to test because there are numerous counter examples in nature in support of either position. For example, a sand pile at a critical slope clearly does not have consciousness, in deep unconscious sleep the brain can be in critical state, and the conscious brain may have non-critical processes. Our findings suggest that the specific aspects of self-organization processes subserving conscious functions are seriously compromised in the brain of patients with UWS. As far as the relevance to consciousness is concerned, the formation of subjective experience has been postulated to depend on widespread information transmission and integration in a global workspace involving large-scale brain networks [63], [64]. Therefore, the investigation of the organization of the whole-brain network and the anatomical-functional parcellation scheme for identifying network nodes can be deemed appropriate for addressing consciousness-related questions. Second, a well-known limitation of fMRI is that it indirectly reflects mass neuronal activity [65]. This defines the hemodynamic nature and possible spatial resolution of functional units (network nodes) that can be derived from the fMRI data. Third, imaging healthy participants undergoing propofol sedation and patients with UWS were performed using different scanners of variant magnetic field strengths. 3.0 Tesla scanning (with UWS patients) has a higher signal-to-noise ratio, resulting in stronger correlation strengths when compared with that obtained with a scanner of 1.5 Tesla (with healthy participant undergoing sedation). This gave rise to an issue on the selection of thresholds when cross-correlation matrices had to be thresholded to estimate node-degree distribution in the two groups. A uniform threshold would incur different degrees of separating effects to the correlation matrices of the two groups because of the differences introduced by scanning parameters, particularly by the magnetic field strength. Meanwhile, too low or too high thresholds would ultimately result in either fully connected or unconnected networks, preventing an assessment of scale-free characteristics. The issue, however, was mitigated by an experimental selection of different thresholds applied to the correlation matrices of the two groups. In our case, the threshold was set at 0.2 for the correlation matrices of healthy participants and at 0.4 of patients with UWS. The selection of the thresholds could result in approximately comparable numbers of remaining connection pairs in the correlation matrices of the two groups. We also verified that varying the thresholds around a small neighborhood of the chosen values would not significantly alter the results. Lastly, the proper statistical method that can be used to validate a power-law scaling in experimental data with mathematical rigor has been controversial [66], [67]. We consider that by comparing power-law fitting with exponential and logarithmic fittings, our approach provides a reasonably well estimation on the issue. We are aware of the maximum likelihood estimator developed by Clauset et al. for evaluating power-law distribution in empirical data [68]. When we applied this approach to our datasets, the goodness-of-fit tests did not show a power-law distribution of node degrees. We speculate that the discrepancy is due to a difference between mathematical reality and biological reality, as often encountered in statistical tests and corrections, such as the correction for multiple comparisons in neuroimaging signal analysis. Given the fact that the formation of the histograms of node size and node degree and the resultant dynamic ranges of the histograms were largely determined by the spatial scale, i.e., the number of network nodes, we did not expect the power-law distribution extend over several orders of magnitude, as found, for example, in neuronal avalanches [16].

In summary, this study reports a fundamental difference in the node-degree distribution of brain network organization between anesthetized healthy participants and patients with UWS. The maintenance of scale-free configuration in healthy brains across the states of general anesthesia, despite profound propofol-induced functional connectivity changes, suggests the presence of a self-organizing process that persists from wakefulness to sedation and recovery. In contrast, the node-degree distribution of patients with UWS shows no scale-free characteristics. This suggests an absence of self-organizing processes for regulating functional interactions among network components, which potentially accounts for the patients' failure to spontaneously regain consciousness. The SOC theory, which motivated this study from the beginning, appears to provide a plausible conceptual framework for the prediction and explanation of findings by the study. Future investigations should extend the findings to examine if the presence or absence of power-law characteristics in the brain's structural and functional network organizations may have predictive value for the recovery of consciousness in brain-injured patients, such as those in coma or minimally conscious state.

## Supporting Information

Figure S1 **Node-size distribution across different spatial scales in one healthy participant (as shown in** **Figure 2** **) in deep sedation.** (**A**) Node-size distribution of the original 116 anatomical nodes. (**B-L**) As network nodes were defined at finer spatial scales, a power-law node-size distribution became increasingly evident. The minimum and maximum node sizes were shown in the subplots.
(TIF)

Figure S2 **Node-size distribution across different spatial scales in one healthy participant (as shown in** **Figure 2** **) in recovery.** (**A**) Node-size distribution of the original 116 anatomical nodes. (**B-L**) As network nodes were defined at finer spatial scales, a power-law node-size distribution became increasingly evident. The minimum and maximum node sizes were shown in the subplots.
(TIF)

Figure S3 **Node-degree distribution in one healthy participant (as shown in** **Figure 4** **) in deep sedation.** (**A**) Node-degree distribution of the original 116 anatomical nodes. (**B-L**) As network nodes were defined at finer spatial scales, a power-law node-degree distribution became increasingly evident. The minimum and maximum node degrees after thresholding were shown in the subplots.
(TIF)

Figure S4 **Node-degree distribution in one healthy participant (as shown in** **Figure 4** **) in recovery.** (**A**) Node-degree distribution of the original 116 anatomical nodes. (**B-L**) As network nodes were defined at finer spatial scales, a power-law node-degree distribution became increasingly evident. The minimum and maximum node degrees after thresholding were shown in the subplots.
(TIF)

Figure S5 **Node-size distribution in one UWS patient (as shown in** **Figure 6** **).** (**A**) Node-size distribution of the original 116 anatomical nodes. (**B-L**) As network nodes were defined at finer spatial scales, a power-law-shaped node-size distribution became increasingly evident. The minimum and maximum node sizes were shown in the subplots.
(TIF)
