# Supplementary figures and images for: Scale-Free Functional Connectivity of the Brain Is Maintained in Anesthetized Healthy Participants but Not in Patients with Unresponsive Wakefulness Syndrome
Source: PLoS One. 2014 Mar 19;9(3):e92182. doi: 10.1371/journal.pone.0092182 (PMC3960221; doi:10.1371/journal.pone.0092182)

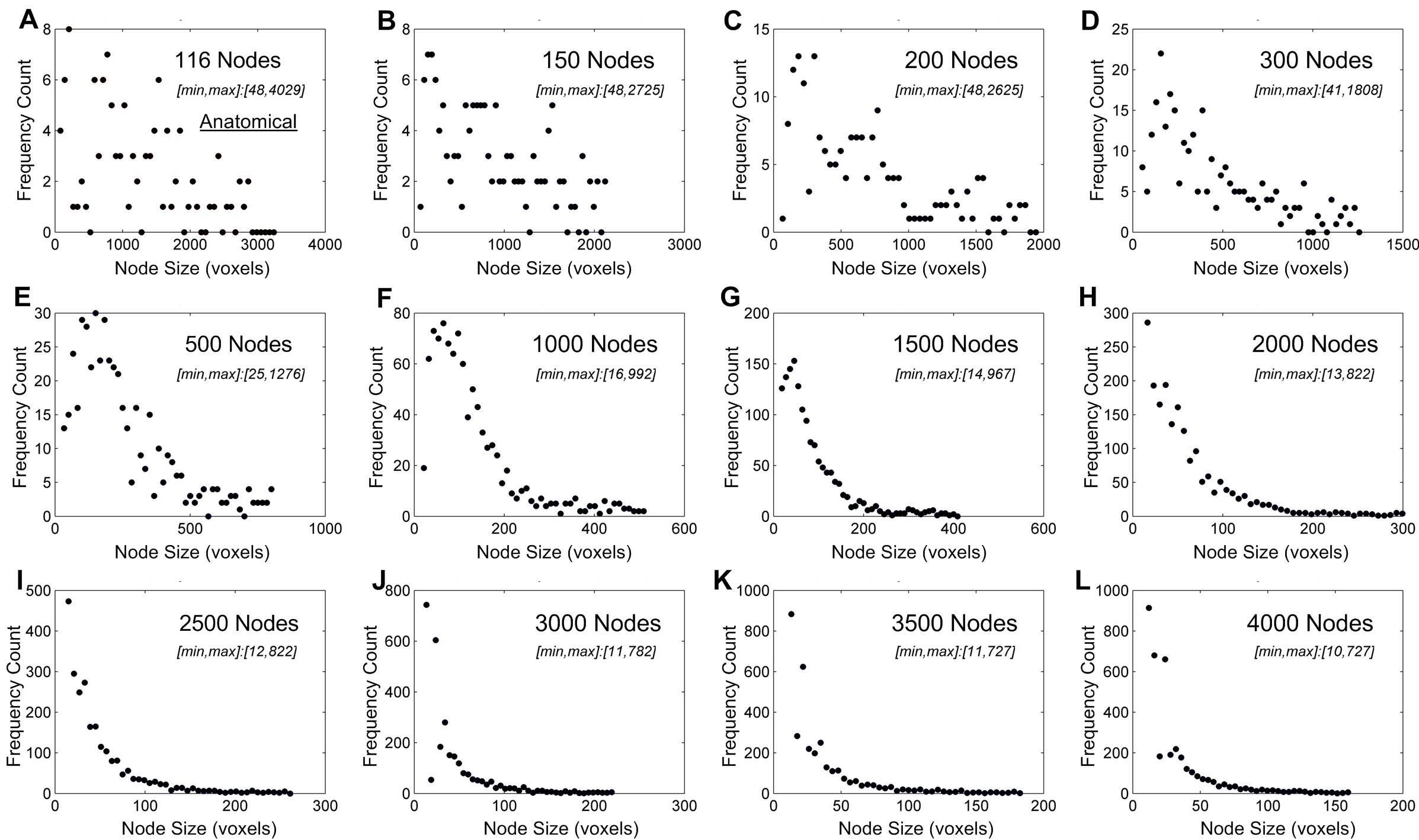

Supplement: Figure S1 — Node-size distribution across different spatial scales in one healthy participant (as shown in Figure 2 ) in deep sedation. (A) Node-size distribution of the original 116 anatomical nodes. (B-L) As network nodes were defined at finer spatial scales, a power-law node-size distribution became increasingly evident. The minimum and maximum node sizes were shown in the subplots. (TIF) [file pone.0092182.s001.tif]

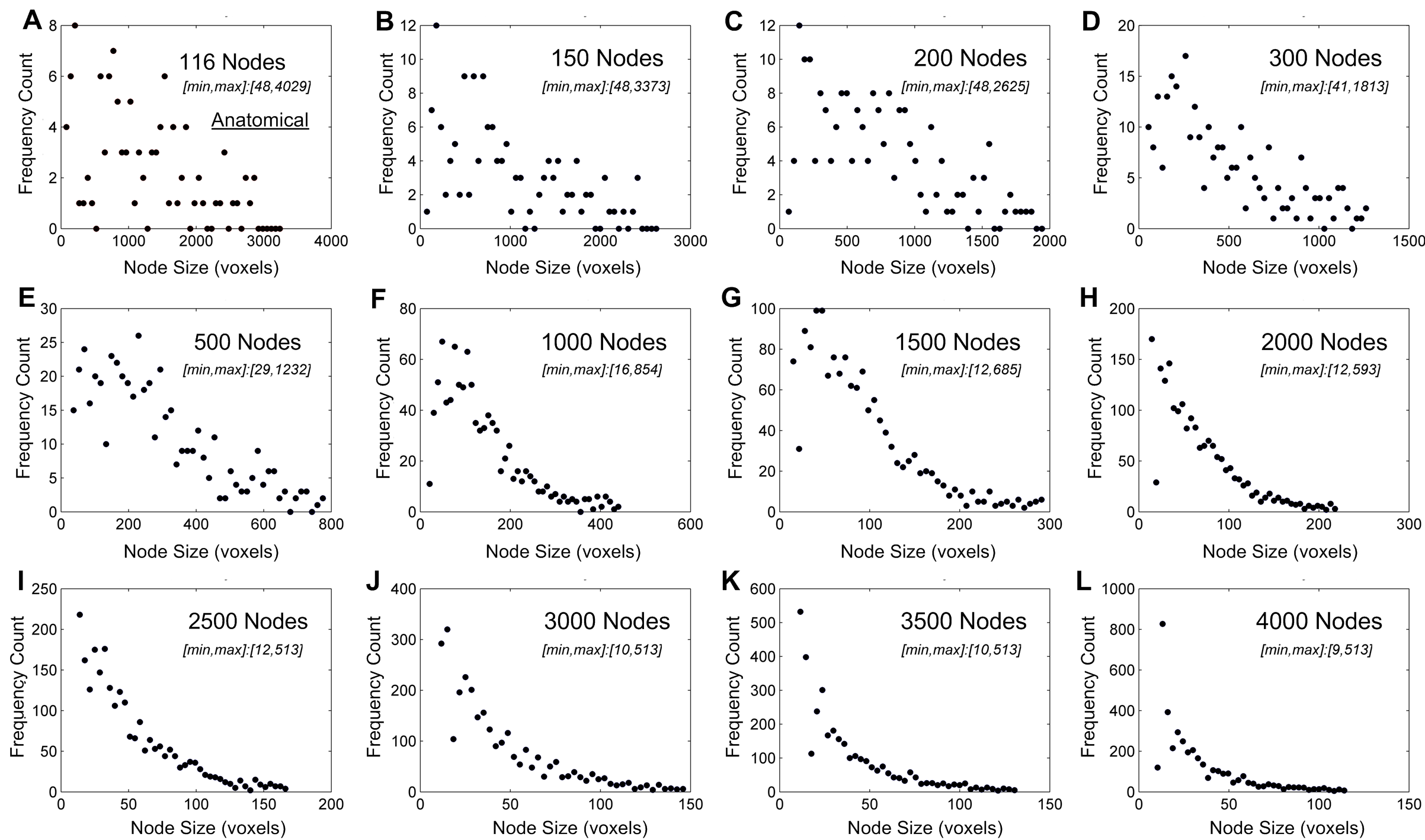

Supplement: Figure S2 — Node-size distribution across different spatial scales in one healthy participant (as shown in Figure 2 ) in recovery. (A) Node-size distribution of the original 116 anatomical nodes. (B-L) As network nodes were defined at finer spatial scales, a power-law node-size distribution became increasingly evident. The minimum and maximum node sizes were shown in the subplots. (TIF) [file pone.0092182.s002.tif]

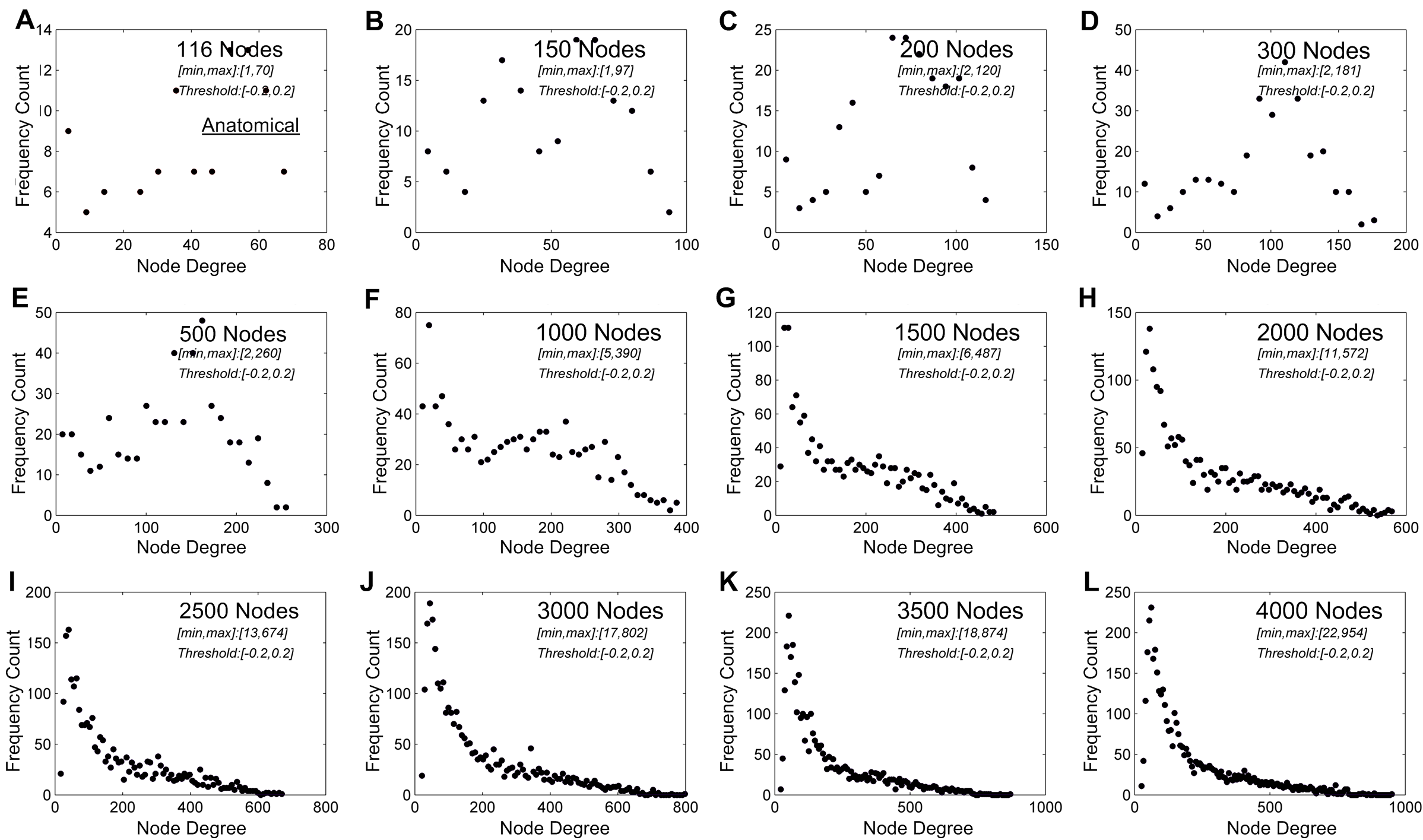

Supplement: Figure S3 — Node-degree distribution in one healthy participant (as shown in Figure 4 ) in deep sedation. (A) Node-degree distribution of the original 116 anatomical nodes. (B-L) As network nodes were defined at finer spatial scales, a power-law node-degree distribution became increasingly evident. The minimum and maximum node degrees after thresholding were shown in the subplots. (TIF) [file pone.0092182.s003.tif]

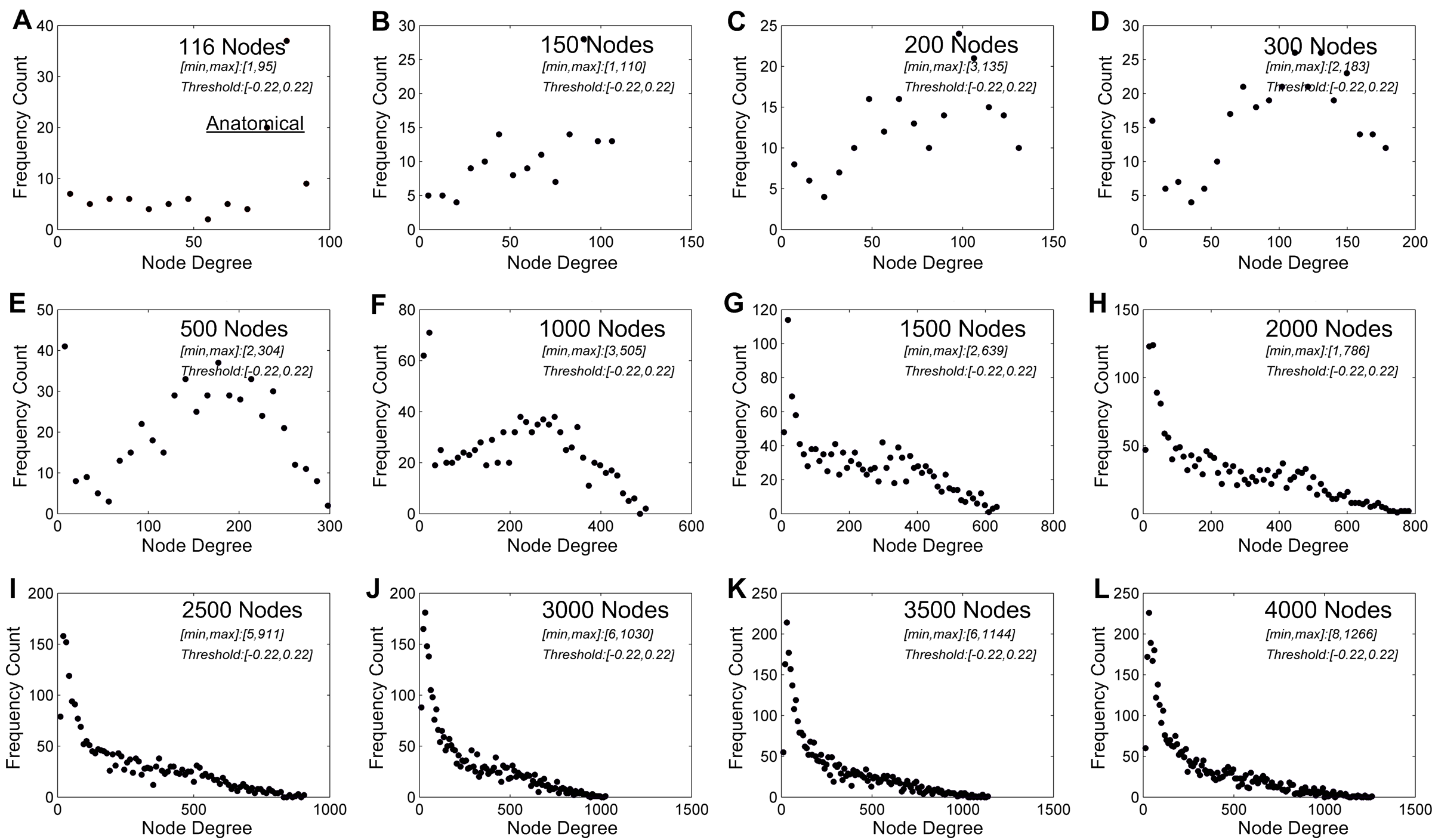

Supplement: Figure S4 — Node-degree distribution in one healthy participant (as shown in Figure 4 ) in recovery. (A) Node-degree distribution of the original 116 anatomical nodes. (B-L) As network nodes were defined at finer spatial scales, a power-law node-degree distribution became increasingly evident. The minimum and maximum node degrees after thresholding were shown in the subplots. (TIF) [file pone.0092182.s004.tif]

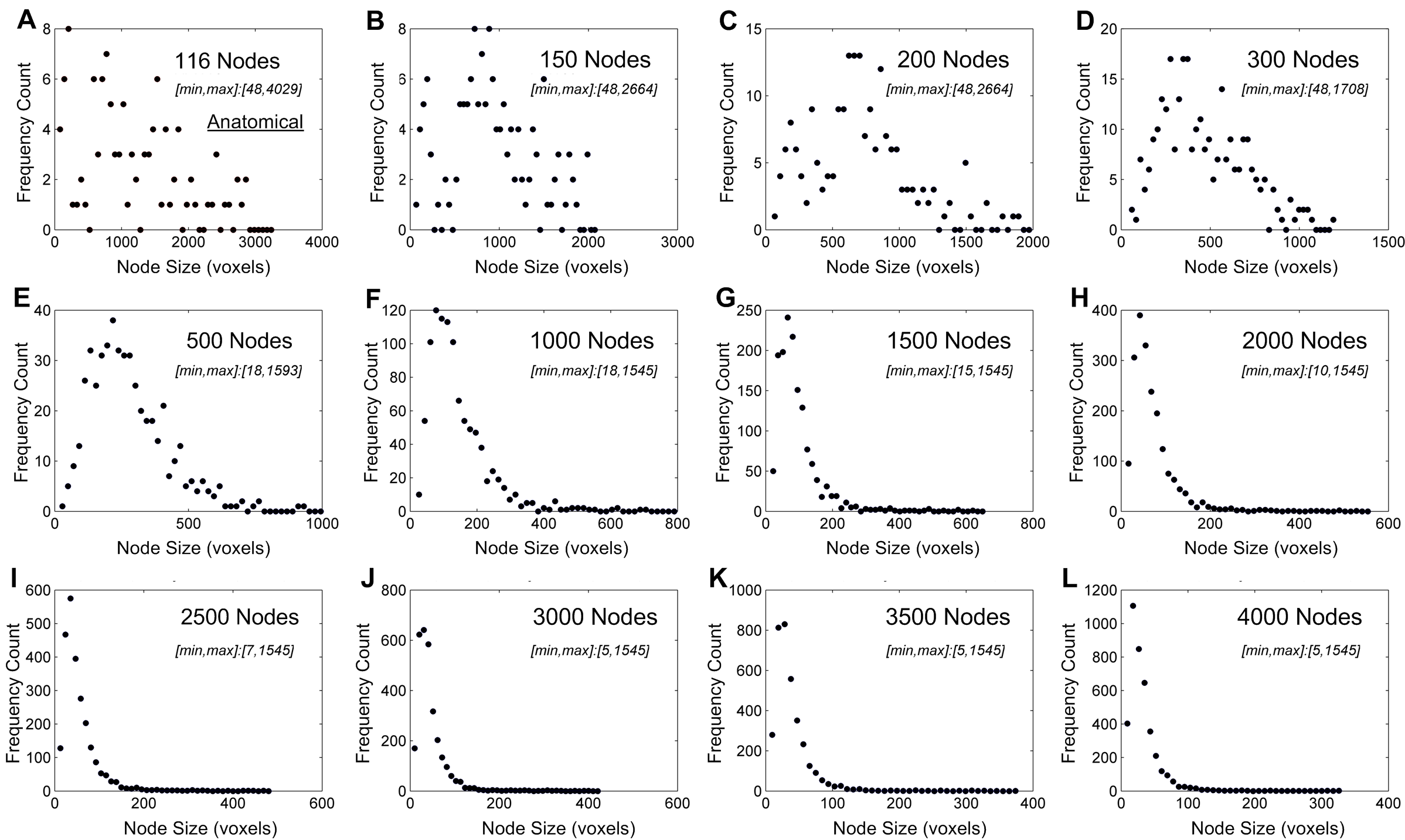

Supplement: Figure S5 — Node-size distribution in one UWS patient (as shown in Figure 6 ). (A) Node-size distribution of the original 116 anatomical nodes. (B-L) As network nodes were defined at finer spatial scales, a power-law-shaped node-size distribution became increasingly evident. The minimum and maximum node sizes were shown in the subplots. (TIF) [file pone.0092182.s005.tif]
